# Supplementary material for: A Novel Predictor Tool of Biochemical Recurrence after Radical Prostatectomy Based on a Five-MicroRNA Tissue Signature
Source: Cancers (Basel). 2019 Oct 21;11(10):1603. doi: 10.3390/cancers11101603 (PMC6826532; doi:10.3390/cancers11101603)
Supplement: Supplementary file 1 [file cancers-11-01603-s001.pdf]

Supplementary Materials

Supplementary Information S1: Sample size and power calculations

The conventional thresholds of α=5% (significance level) and β=20% (1-power; power of 80%) were applied for sample size calculation using the statistics programs MedCalc 19.0.6 (MedCalc Software bvba, Ostend, Belgium), GPower 3.1.9.4 (Franz Faul; Kiel University, Kiel, Germany), and GraphPad StatMate 2.0 (GraphPad Software, San Diego, CA, USA).

Receiver-operating characteristics (ROC) analysis was performed to calculate the necessary sample size to evaluate the capacity of the different miRNAs to discriminate between matched pairs of malignant and adjacent normal tissue. According to previous studies, we defined as appropriate discrimination an area under the ROC curve higher than 0.75. A sample size of at least 19 pairs would be necessary.

The necessary sample size was calculated for assessing the usefulness of variables/markers for the clinical prognostic endpoint of biochemical recurrence (BCR). Assuming a difference of 0.20 in the Kaplan-Meier "survival" rate within five-year follow-up after radical prostatectomy and selecting tissue samples in this retrospective study by an alternate selection process of patients with (assuming a rate of 0.70) and without BCR (assuming a rate of 0.90) in a consecutive manner resulting in a ratio of one, a total sample size of at least 67 would be required.

Table S1. TaqMan MicroRNA assays used for RT-qPCR analyses Assay names and assay IDs are taken from the nomenclature of the supplier Thermo Fisher. The miRBase accession no., miRBase ID, target sequences, gene family data, and clustered miRNAs were taken from the miRBase database release 22 (Sanger Institute, Manchester, UK; http://www.mirbase.org).

miRBase ID Release 22	miRBase Accession No.	AB Assay Name	AB Assay ID	Sequence	Chromosome Location/Coordinates on Build GRCh38	miRNA Gene Family	Clustered miRNAs
hsa-miR-1-3p	MIMAT0000416	hsa-miR-1	002222	UGGAAUGUAAAGA AGUAUGUAU	Chr. 18: 21829004 - 21829088 [-]	mir-1 (mir-1-1, mir-1-2, mir-206)	mir-133a-1
hsa-miR-21-5p	MIMAT0000076	hsa-miR-21	000397	UAGCUUAUCAGAC UGAUGUUGA	Chr. 17: 59841266 - 59841337 [+]	mir-21	–
hsa-miR-29c-3p	MIMAT0000681	hsa-miR-29c	000587	UAGCACCAUUUGA AAUCGGUUA	Chr. 1: 207801852 - 207801939 [-]	mir-29 (mir-29a, mir-29b-1, mir-29b-2,mir-29c)	mir-29b-2
hsa-miR-30c-5p	MIMAT0000244	hsa-miR-30c	000419	UGUAAACAUCCUAC ACUCUCAGC	Chr. 1: 40757284 - 40757372 [+]	mir-30 (mir-30a, mir-30b, mir-30c-1, mir-30c-2, mir-30d, mir-30e)	–
hsa-miR-30d-5p	MIMAT0000245	hsa-miR-30d	000420	UGUAAACAUCCCCG ACUGGAAG	Chr. 8: 134804876 - 134804945 [-]	mir-30 (mir-30a, mir-30b, mir-30c-1, mir-30c-2, mir-30d, mir-30e)	mir-30b
hsa-miR-31-5p	MIMAT0000089	hsa-miR-31	002279	AGGCAAGAUGCUG GCAUAGCU	Chr. 9: 21512115 - 21512185 [-]	mir-31	–
hsa-miR-34a-5p	MIMAT0000255	hsa-miR-34a	000426	UGGCAGUGUCUUA GCUGGUUGU	Chr. 1: 9151668 -9151777 [-]	mir-34 (mir-34a, mir-34b, mir-34c)	–
hsa-miR-141-3p	MIMAT0000432	hsa-miR-141	000463	UAACACUGUCUGG UAAAGAUGG	Chr.12: 6964097 - 6964191 [+]	mir-8 (mir-141, mir-200a, mir-200b, mir-200c, mir-429)	mir-200c
hsa-miR-145-5p	MIMAT0000437	hsa-miR-145	002278	GUCCAGUUUUCCCA GGAAUCCCU	Chr. 5: 149430646 - 149430733 [+]	mir 145	mir-143
hsa-miR-148a-3p	MIMAT0000243	hsa-miR-148a	000470	UCAGUGCACUACAG AACUUUGU	Chr. 7: 25949919 - 25949986 [-]	mir-148 (mir-148a, mir-148b, mir-152)	–
hsa-miR-185-5p	MIMAT0000455	hsa-miR-185	002271	UGGAGAGAAAGGC AGUUCCUGA	Chr. 22: 20033139 - 20033220 [+]	mir-185	–
hsa-miR-195-5p	MIMAT0000461	hsa-miR-195	000494	UAGCAGCACAGAA AUAUUGGC	Chr. 17: 7017615 - 7017701 [-]	mir-15 (mir-15a, mir-15b, mir-16-1, mir-16-2, mir-195)	mir-497
hsa-miR-204-5p	MIMAT0000265	hsa-miR-204	000508	UUCCCUUUGUCAUC CUAUGCCU	Chr. 9: 70809975 - 70810084 [-]	mir-204 (mir-204, mir-211)	–

miRBase ID Release 22	miRBase Accession No.	AB Assay Name	AB Assay ID	Sequence	Chromosome Location/Coordinates on Build GRCh38	miRNA Gene Family	Clustered miRNAs
hsa-miR-221-3p	MIMAT0000278	hsa-miR-221	000524	AGCUACAUUGUCU GCUGGGUUUC	Chr. X: 45746157 - 45746266 [-]	mir-221 (mir-221, mir-222)	mir-222
hsa-miR-224-5p +	MIMAT0000281	hsa-miR-224	002099	UCAAGUCACUAGU GGUUCCGUUUAG	Chr. X: 151958578 - 151958658 [-]	mir-224	mir-452
hsa-miR-301a-3p	MIMAT0000688	hsa-miR-301	000528	CAGUGCAAUAGUA UUGUCAAAGC	Chr. 17: 59151136 - 59151221 [-]	mir-130 (mir-130a, mir-130b, mir-301a, mir-301b)	–
hsa-miR-326	MIMAT0000756	hsa-miR-326	000542	CCUCUGGGCCCUUC CUCCAG	Chr. 11: 7533509 - 75335186 [-]	mir-326	–
hsa-miR-374b-5p	MIMAT0004955	hsa-miR-374b-5p	001319	AUAUAAUACAACC UGCUAAGUG	Chr. X: 74218547 - 74218618 [-]	mir-374 (mir-374a, mir-374b, mir-374c)	–
hsa-miR-494-3p	MIMAT0002816	hsa-miR-494	002365	UGAAACAUACACG GGAAACCUC	Chr. 14: 101029634 - 101029714 [+]	mir-154 (mir-154, mir-300, mir-323a, mir-323b, mir-369, mir-377, mir-381, mir-382, mir-409, mir-410, mir-487a, mir-487b, mir-494, mir-496, mir-539, mir-655, mir-656, mir-1185-1, mir-1185-2)	mir-299, mir-323a, mir-329-1, mir-329-2, mir-379, mir-380, mir-411, mir-494, mir-495, mir-543, mir-758, mir-1193, mir-1197
hsa-miR-939-5p	MIMAT0004982	hsa-miR-939	002182	UGGGGAGCUGAGG CUCUGGGGGUG	Chr. 8: 144394149 - 144394230 [-]	mir-939	mir-1234, mir-6849
**Reference-miRNAs**							
hsa-let-7g-5p	MIMAT0000414	hsa-let-7g	002282	UGAGGUAGUAGUU UGUACAGUU	Chr. 3: 52268278 - 52268361 [-]	let7 (let-7a-1, let-7a-2, let-7a-3, let-7b, let-7c, let-7d, let-7e, let-7f-1, let-7f-2, mir-98, let-7g, let-7i)	–
hsa-miR-103a-3p	MIMAT0000101	hsa-miR-103	000439	AGCAGCAUUGUAC AGGGCUAUGA	Chr. 5: 168560896 - 168560973 [-]	mir-103 (mir-103a-1, mir-103a-2, mir-103b-1, mir-103b-2, mir-107)	mir-103b-2

+ Until miRBase 21; however, this miRNA has now the miRBase ID eca-miR-224 (accession no. MIMAT0013206).

Table S2. MicroRNAs in tumor samples in relationship to the digital rectal examination result.

Variable	DRE = non-suspicious			DRE = suspicious			
	n	Median	Average Rank	n	Median	Average Rank	*p* value [a]
miR-1-3p	122	1.0850	109.3934	84	0.8950	94.9405	0.087
miR-21-5p	122	0.9700	97.0041	84	1.0850	112.9345	0.059
miR-29c-3p	122	0.9900	105.2049	84	0.8500	101.0238	0.621
miR-30c-5p	122	1.0650	109.7828	84	0.9750	94.3750	0.068
miR-30d-5p	122	0.9450	102.4672	84	0.9900	105.0000	0.764
miR-31-5p	122	1.3800	106.1352	84	1.0550	99.6726	0.445
miR-34a-5p	122	0.9650	105.0369	84	0.9200	101.2679	0.656
miR-141-3p	122	1.0300	104.5697	84	0.9750	101.9464	0.756
miR-145-5p	122	1.0250	106.5615	84	0.9700	99.0536	0.374
miR-148a-3p	122	1.0350	103.2623	84	0.9700	103.8452	0.945
miR-185-5p	122	0.9750	102.8893	84	0.9550	104.3869	0.859
miR-195-5p	122	1.0450	101.8525	84	1.1000	105.8929	0.633
miR-204-5p	122	1.0550	106.5779	84	0.9200	99.0298	0.372
miR-221-3p	122	0.7950	107.5246	84	0.6950	97.6548	0.243
miR-224-5p	122	0.9050	105.3730	84	0.8850	100.7798	0.587
miR-301a-3p	122	1.0500	103.4549	84	1.0200	103.5655	0.990
miR-326	122	1.0100	103.5246	84	1.0050	103.4643	0.994
miR-374b-5p	122	1.0750	105.1557	84	1.0200	101.0952	0.631
miR-494-3p	122	0.8050	100.0000	84	0.8300	108.5833	0.310
miR-939-5p	122	0.9850	103.6475	84	1.0300	103.2857	0.966

Abbreviations: DRE, digital rectal examination, 0=non-suspicious, 1=suspicious. [a] Mann-Whitney test.

Table S3. MicroRNAs in tumor samples in relationship to the margin status.

Variable	pN status = negative			pN status = positive			
	n	Median	Average Rank	n	Median	Average Rank	*p*-value [a]
miR-1-3p	196	1.0500	104.9770	10	0.6550	74.5500	0.115
miR-21-5p	196	1.0150	103.2041	10	1.1900	109.3000	0.752
miR-29c-3p	196	0.9800	105.4796	10	0.6100	64.7000	0.035
miR-30c-5p	196	1.0250	105.1403	10	0.8100	71.3500	0.080
miR-30d-5p	196	0.9900	104.7194	10	0.7200	79.6000	0.194
miR-31-5p	196	1.3300	105.2372	10	0.2750	69.4500	0.064
miR-34a-5p	196	0.9500	105.4107	10	0.6750	66.0500	0.042
miR-141-3p	196	1.0150	104.1224	10	0.8950	91.3000	0.507
miR-145-5p	196	0.9900	104.9821	10	0.6650	74.4500	0.114
miR-148a-3p	196	1.0150	103.4362	10	1.0000	104.7500	0.946
miR-185-5p	196	0.9650	103.7704	10	1.0750	98.2000	0.773
miR-195-5p	196	1.0700	103.0842	10	1.2300	111.6500	0.658
miR-204-5p	196	1.0300	104.9745	10	0.8200	74.6000	0.116
miR-221-3p	196	0.7500	104.1403	10	0.7050	90.9500	0.495
miR-224-5p	196	0.8950	104.9184	10	0.5700	75.7000	0.131
miR-301a-3p	196	1.0350	103.0485	10	1.1800	112.3500	0.630
miR-326	196	1.0100	104.5689	10	0.8300	82.5500	0.255
miR-374b-5p	196	1.0600	104.8010	10	0.8000	78.0000	0.166
miR-494-3p	196	0.8050	103.9158	10	0.8750	95.3500	0.658
miR-939-5p	196	1.0250	105.2500	10	0.6750	69.2000	0.062

Abbreviations: pN status, lymph node status. [a] Mann-Whitney test, significant values ($p < 0.050$) are highlighted in yellow.

Table S4. MicroRNAs in tumor samples in relationship to the lymph node status.

Variable	pN status = negative			pN status = positive			
	n	Median	Average Rank	n	Median	Average Rank	*p*-value [a]
miR-1-3p	196	1.0500	104.9770	10	0.6550	74.5500	0.115
miR-21-5p	196	1.0150	103.2041	10	1.1900	109.3000	0.752
miR-29c-3p	196	0.9800	105.4796	10	0.6100	64.7000	0.035
miR-30c-5p	196	1.0250	105.1403	10	0.8100	71.3500	0.080
miR-30d-5p	196	0.9900	104.7194	10	0.7200	79.6000	0.194
miR-31-5p	196	1.3300	105.2372	10	0.2750	69.4500	0.064
miR-34a-5p	196	0.9500	105.4107	10	0.6750	66.0500	0.042
miR-141-3p	196	1.0150	104.1224	10	0.8950	91.3000	0.507
miR-145-5p	196	0.9900	104.9821	10	0.6650	74.4500	0.114
miR-148a-3p	196	1.0150	103.4362	10	1.0000	104.7500	0.946
miR-185-5p	196	0.9650	103.7704	10	1.0750	98.2000	0.773
miR-195-5p	196	1.0700	103.0842	10	1.2300	111.6500	0.658
miR-204-5p	196	1.0300	104.9745	10	0.8200	74.6000	0.116
miR-221-3p	196	0.7500	104.1403	10	0.7050	90.9500	0.495
miR-224-5p	196	0.8950	104.9184	10	0.5700	75.7000	0.131
miR-301a-3p	196	1.0350	103.0485	10	1.1800	112.3500	0.630
miR-326	196	1.0100	104.5689	10	0.8300	82.5500	0.255
miR-374b-5p	196	1.0600	104.8010	10	0.8000	78.0000	0.166

miR-494-3p	196	0.8050	103.9158	10	0.8750	95.3500	0.658
miR-939-5p	196	1.0250	105.2500	10	0.6750	69.2000	0.062

Abbreviations: pN status, lymph node status. [a] Mann-Whitney test, significant values (p <0.050) are highlighted in yellow.

Table S5. Associations between clinicopathological variables and microRNAs in the examined tumor samples. The relationships are indicated as p-values calculated by Spearman rank correlation coefficients, Mann-Whitney test or Kruskal-Wallis test.

miRNA	Age [a]	PSA [a]	DRE [b]	Margin [b]	pN status [b]	pT stage [c]	ISUP [c]
miR-1-3p	0.557	0.001 ↓	0.087	0.037 ↓	0.115	0.035	<0.0001
miR-21-5p	0.214	0.922	0.059	0.065	0.752	0.259	0.014
miR-29c-3p	0.139	0.668	0.621	0.424	0.035 ↓	0.970	0.047
miR-30c-5p	0.901	0.125	0.068	0.047 ↓	0.080	0.134	0.642
miR-30d-5p	0.294	0.783	0.764	0.357	0.194	0.852	0.095
miR-31-5p	0.178	0.220	0.445	0.005 ↓	0.064	0.770	0.001
miR-34a-5p	0.318	0.090	0.656	0.102	0.042 ↓	0.261	0.170
miR-141-3p	0.174	0.025 ↑	0.756	0.108	0.507	0.224	0.001
miR-145-5p	0.604	0.002 ↓	0.374	0.009 ↓	0.114	0.098	0.001
miR-148a-3p	0.546	0.334	0.945	0.009 ↑	0.946	0.383	0.001
miR-185-5p	0.976	0.371	0.859	0.168	0.773	0.505	0.481
miR-195-5p	0.870	0.050 ↓	0.633	0.002 ↓	0.658	0.350	0.799
miR-204-5p	0.090	0.002 ↓	0.372	0.076	0.116	0.562	0.002
miR-221-3p	0.983	0.253	0.243	0.651	0.495	0.127	0.004
miR-224-5p	0.208	0.049 ↓	0.587	0.012 ↓	0.131	0.186	0.102
miR-301a-3p	0.528	0.670	0.990	0.253	0.630	0.254	0.441
miR-326	0.517	0.495	0.994	0.186	0.255	0.912	0.138
miR-374b-5p	0.611	0.367	0.631	0.168	0.166	0.826	0.713
miR-494-3p	0.584	0.945	0.310	0.069	0.658	0.287	0.438
miR-939-5p	0.999	0.702	0.966	0.671	0.062	0.088	0.031

Abbreviations: miRNA, human microRNAs, see Table 2; PSA, prostate specific antigen; DRE, digital rectal examination result, positive/negative; pN status, pathological lymph node status, positive/negative; pT stage, pathological tumor classification, see Table 1; ISUP, histopathological grade system based on Gleason score according to the International Society of Urologic Pathology, see Table 1. [a, b, c] p values highlighted in yellow ([a] Spearman rank for age and PSA; [b] Mann-Whitney test for DRE, margin, and pN status; [c] Kruskal-Wallis test for pT stage and ISUP grade groups) indicate significant associations between clinicopathological variables and corresponding miRNAs, details are shown in Tables S2 to S4 and Figures S2 and S3. The arrows indicate positive↑/negative ↓ correlations/associations. The relationships between pT stages and ISUP grades are shown in the Figures S2 and S3.

Table S6. Spearman rank correlation coefficients among the microRNAs analyzed in tumor samples. Significant coefficients (p<0.05) are highlighted in yellow, coefficients >0.5 are highlighted in green.

		**A) miR-1 to miR-148a**									
		**miR-1**	**miR-21**	**miR-29c**	**miR-30c**	**miR-30d**	**miR-31**	**miR-34a**	**miR-141**	**miR-145**	**miR-148a**
**miR-1**	r_s		0.227	0.175	0.420	0.232	0.222	0.358	-0.242	0.856	-0.162
	p value		0.001	0.012	<0.0001	0.001	0.002	<0.0001	0.001	<0.0001	0.021
**miR-21**	r_s	0.227		0.470	0.324	0.429	0.279	0.567	0.311	0.319	0.302
	p value	0.001		<0.0001	<0.0001	<0.0001	<0.0001	<0.0001	<0.0001	<0.0001	<0.0001
**miR-29c**	r_s	0.175	0.470		0.519	0.585	0.027	0.533	0.649	0.254	0.623
	p value	0.012	<0.0001		<0.0001	<0.0001	0.698	<0.0001	<0.0001	0.0002	<0.0001
**miR-30c**	r_s	0.420	0.324	0.519		0.713	0.087	0.526	0.287	0.489	0.310
	p value	<0.0001	<0.0001	<0.0001		<0.0001	0.213	<0.0001	<0.0001	<0.0001	<0.0001
**miR-30d**	r_s	0.232	0.429	0.585	0.713		0.033	0.509	0.416	0.328	0.425
	p value	0.001	<0.0001	<0.0001	<0.0001		0.634	<0.0001	<0.0001	<0.0001	<0.0001
**miR-31**	r_s	0.222	0.279	0.027	0.087	0.033		0.209	-0.069	0.230	-0.162
	p value	0.002	<0.0001	0.698	0.213	0.634		0.003	0.326	0.001	0.020
**miR-34a**	r_s	0.358	0.567	0.533	0.526	0.509	0.209		0.307	0.526	0.314
	p value	<0.0001	<0.0001	<0.0001	<0.0001	<0.0001	0.003		<0.0001	<0.0001	<0.0001
**miR-141**	r_s	-0.242	0.311	0.649	0.287	0.416	-0.069	0.307		-0.137	0.752
	p value	0.001	<0.0001	<0.0001	<0.0001	<0.0001	0.326	<0.0001		0.050	<0.0001
**miR-145**	r_s	0.856	0.319	0.254	0.489	0.328	0.230	0.526	-0.137		-0.118
	p value	<0.0001	<0.0001	0.000	<0.0001	<0.0001	0.001	<0.0001	0.050		0.090
**miR-148a**	r_s	-0.162	0.302	0.623	0.310	0.425	-0.162	0.314	0.752	-0.118	
	p value	0.021	<0.0001	<0.0001	<0.0001	<0.0001	0.020	<0.0001	<0.0001	0.090	
**miR-185**	r_s	0.282	0.652	0.469	0.540	0.639	0.118	0.648	0.274	0.397	0.215
	p value	<0.0001	<0.0001	<0.0001	<0.0001	<0.0001	0.092	<0.0001	0.0001	<0.0001	0.002
**miR-195**	r_s	0.385	0.480	0.295	0.616	0.553	0.089	0.435	-0.015	0.495	0.059
	p value	<0.0001	<0.0001	<0.0001	<0.0001	<0.0001	0.204	<0.0001	0.825	<0.0001	0.399
**miR-204**	r_s	0.579	0.161	0.126	0.322	0.164	0.154	0.461	-0.188	0.712	-0.197
	p value	<0.0001	0.021	0.072	<0.0001	0.018	0.028	<0.0001	0.007	<0.0001	0.005
**miR-221**	r_s	0.553	0.315	0.342	0.229	0.159	0.152	0.515	0.119	0.658	0.150
	p value	<0.0001	<0.0001	<0.0001	0.001	0.023	0.029	<0.0001	0.087	<0.0001	0.031
**miR-224**	r_s	0.548	0.338	0.146	0.236	0.105	0.578	0.376	-0.086	0.631	-0.145
	p value	<0.0001	<0.0001	0.037	0.001	0.135	<0.0001	<0.0001	0.220	<0.0001	0.038
**miR-301a**	r_s	0.479	0.418	0.228	0.537	0.475	-0.059	0.420	-0.103	0.517	0.037
	p value	<0.0001	<0.0001	0.001	<0.0001	<0.0001	0.401	<0.0001	0.139	<0.0001	0.595

**miR-326**	r_s	0.334	0.454	0.454	0.560	0.706	0.125	0.668	0.221	0.414	0.204
	p value	<0.0001	<0.0001	<0.0001	<0.0001	<0.0001	0.073	<0.0001	0.001	<0.0001	0.003
**miR-374b**	r_s	0.564	0.484	0.599	0.743	0.667	0.077	0.556	0.291	0.613	0.332
	p value	<0.0001	<0.0001	<0.0001	<0.0001	<0.0001	0.272	<0.0001	<0.0001	<0.0001	<0.0001
**miR-494**	r_s	0.208	0.204	0.194	-0.086	0.129	-0.066	0.180	0.149	0.219	0.189
	p value	0.003	0.003	0.005	0.217	0.064	0.348	0.010	0.032	0.002	0.007
**miR-939**	r_s	0.146	0.041	0.087	0.031	0.152	0.122	0.029	-0.031	0.066	0.029
	p value	0.038	0.559	0.214	0.660	0.029	0.080	0.679	0.659	0.343	0.675

B) miR-185 to miR-939

		**miR-185**	**miR-195**	**miR-204**	**miR-221**	**miR-224**	**miR-301a**	**miR-326**	**miR-374b**	**miR-494**	**miR-939**
**miR-1**	r_s	0.282	0.385	0.579	0.553	0.548	0.479	0.334	0.564	0.208	0.146
	p value	<0.0001	<0.0001	<0.0001	<0.0001	<0.0001	<0.0001	<0.0001	<0.0001	0.003	0.038
**miR-21**	r_s	0.652	0.480	0.161	0.315	0.338	0.418	0.454	0.484	0.204	0.041
	p value	<0.0001	<0.0001	0.021	<0.0001	<0.0001	<0.0001	<0.0001	<0.0001	0.003	0.559
**miR-29c**	r_s	0.469	0.295	0.126	0.342	0.146	0.228	0.454	0.599	0.194	0.087
	p value	<0.0001	<0.0001	0.072	<0.0001	0.037	0.001	<0.0001	<0.0001	0.005	0.214
**miR-30c**	r_s	0.540	0.616	0.322	0.229	0.236	0.537	0.560	0.743	-0.086	0.031
	p value	<0.0001	<0.0001	<0.0001	0.001	0.001	<0.0001	<0.0001	<0.0001	0.217	0.660
**miR-30d**	r_s	0.639	0.553	0.164	0.159	0.105	0.475	0.706	0.667	0.129	0.152
	p value	<0.0001	<0.0001	0.018	0.023	0.135	<0.0001	<0.0001	<0.0001	0.064	0.029
**miR-31**	r_s	0.118	0.089	0.154	0.152	0.578	-0.059	0.125	0.077	-0.066	0.122
	p value	0.092	0.204	0.028	0.029	<0.0001	0.401	0.073	0.272	0.348	0.080
**miR-34a**	r_s	0.648	0.435	0.461	0.515	0.376	0.420	0.668	0.556	0.180	0.029
	p value	<0.0001	<0.0001	<0.0001	<0.0001	<0.0001	<0.0001	<0.0001	<0.0001	0.010	0.679
**miR-141**	r_s	0.274	-0.015	-0.188	0.119	-0.086	-0.103	0.221	0.291	0.149	-0.031
	p value	0.0001	0.825	0.007	0.087	0.220	0.139	0.001	<0.0001	0.032	0.659
**miR-145**	r_s	0.397	0.495	0.712	0.658	0.631	0.517	0.414	0.613	0.219	0.066
	p value	<0.0001	<0.0001	<0.0001	<0.0001	<0.0001	<0.0001	<0.0001	<0.0001	0.002	0.343
**miR-148a**	r_s	0.215	0.059	-0.197	0.150	-0.145	0.037	0.204	0.332	0.189	0.029
	p value	0.002	0.399	0.005	0.031	0.038	0.595	0.003	<0.0001	0.007	0.675
**miR-185**	r_s		0.597	0.282	0.270	0.251	0.602	0.763	0.619	0.213	0.208
	p value		<0.0001	<0.0001	0.0001	0.0003	<0.0001	<0.0001	<0.0001	0.002	0.003
**miR-195**	r_s	0.597		0.356	0.113	0.214	0.662	0.491	0.637	0.036	0.147
	p value	<0.0001		<0.0001	0.106	0.002	<0.0001	<0.0001	<0.0001	0.610	0.035
**miR-204**	r_s	0.282	0.356		0.503	0.441	0.381	0.298	0.348	0.189	0.060
	p value	<0.0001	<0.0001		<0.0001	<0.0001	<0.0001	<0.0001	<0.0001	0.007	0.393

**miR-221**	r_s	0.270	0.113	0.503		0.544	0.290	0.277	0.375	0.272	-0.105
	p value	0.0001	0.106	<0.0001		<0.0001	<0.0001	0.0001	<0.0001	0.0001	0.135
**miR-224**	r_s	0.251	0.214	0.441	0.544		0.158	0.220	0.322	0.136	0.052
	p value	0.0003	0.002	<0.0001	<0.0001		0.023	0.002	<0.0001	0.052	0.458
**miR-301a**	r_s	0.602	0.662	0.381	0.290	0.158		0.564	0.565	0.055	0.110
	p value	<0.0001	<0.0001	<0.0001	<0.0001	0.023		<0.0001	<0.0001	0.433	0.115
**miR-326**	r_s	0.763	0.491	0.298	0.277	0.220	0.564		0.632	0.224	0.265
	p value	<0.0001	<0.0001	<0.0001	0.0001	0.002	<0.0001		<0.0001	0.001	0.000
**miR-374b**	r_s	0.619	0.637	0.348	0.375	0.322	0.565	0.632		0.193	0.158
	p value	<0.0001	<0.0001	<0.0001	<0.0001	<0.0001	<0.0001	<0.0001		0.006	0.023
**miR-494**	r_s	0.213	0.036	0.189	0.272	0.136	0.055	0.224	0.193		0.444
	p value	0.002	0.610	0.007	0.0001	0.052	0.433	0.001	0.006		<0.0001
**miR-939**	r_s	0.208	0.147	0.060	-0.105	0.052	0.110	0.265	0.158	0.444	
	p value	0.003	0.035	0.393	0.135	0.458	0.115	0.0001	0.023	<0.0001	

Abbreviations: miR, microRNA in its abbreviated form to facilitate the readability of the table; full annotation in Table 2; r_s, Spearman rank correlation coefficient.

Table S7. Construction of a miRNA-based predictive classifier for biochemical recurrence using sample splitting approach. The 140 samples of the training set were randomly selected. The test set (n=66) was used to control the final model obtained with the training set (see Table 5).

**miRNA**	**Univariate Cox Regression with a Training Set of n = 140**		**Multivariate Cox Regression with Significant Univariate miRs** [a]			
			**Full model**		**Backward elimination**	
	**HR (95% CI)**	***p*-value**	**HR (95% CI)**	**p-value**	**HR (95% CI)**	***p*-value**
miR-1-3p	0.67 (0.48 - 0.95)	0.026	0.92 (0.53-1.61)	0.774		
miR-21-5p	1.12 (1.01 - 1.24)	0.029	1.48 (0.85-2.59)	0.170		
miR-29c-3p	1.08 (0.81 - 1.45)	0.599				
miR-30c-5p	0.57 (0.31 - 1.06)	0.077	0.32 (0.12-0.86)	0.023	0.52 (0.28-0.96)	0.038
miR-30d-5p	1.25 (1.08 - 1.44)	0.003	1.82 (1.07-3.09)	0.028		
miR-31-5p	0.75 (0.62 - 0.90)	0.002	0.76 (0.61-0.95)	0.017	0.71 (0.58-0.87)	0.001
miR-34a-5p	1.12 (1.00 - 1.24)	0.043	0.55 (0.28-1.09)	0.087		
miR-141-3p	1.23 (1.08 - 1.42)	0.003	3.58 (1.66-7.70)	0.001	2.59 (1.90-4.60)	<0.0001
miR-145-5p	0.75 (0.54 - 1.05)	0.091	1.71 (0.85-3.43)	0.131		
miR-148a-3p	1.14 (0.97 - 1.34)	0.114	0.44 (0.23-0.87)	0.018	0.50 (0.34-0.74)	<.0001
miR-185-5p	1.06 (1.01 - 1.12)	0.019	0.83 (0.50-1.40)	0.489		
miR-195-5p	0.96 (0.66 - 1.39)	0.831				
miR-204-5p	0.83 (0.62 - 1.11)	0.204				
miR-221-3p	0.86 (0.69 - 1.07)	0.164	0.53 (0.32-0.88)	0.014	0.66 (0.52-0.85)	0.001
miR-224-5p	0.71 (0.49 - 1.03)	0.072	0.80 (0.43-1.46)	0.457		
miR-301a-3p	0.91 (0.64 - 1.30)	0.609				
miR-326	1.03 (1.01 - 1.05)	0.007	1.01 (0.79-1.29)	0.918		
miR-374b-5p	0.91 (0.49 - 1.69)	0.770				
miR-494-3p	1.00 (1.00 - 1.01)	0.025	1.01 (0.97-1.04)	0.731		
miR-939-5p	1.02 (1.00 - 1.05)	0.022	0.92 (0.77-1.30)	0.999		

Abbreviations: HR, hazard ratio; CI, confidence interval.

Table S8. C-statistics of three predictive BCR models based on only clinicopathological factors in comparison to 5-miR-panels and their combination. Data are given as ΔAUCs with *p* values in parentheses between the respective models of each group. Abbreviation: BCR, biochemical recurrence.

**Model**	**D'Amico-Based Models**		**CAPRAS-Based Model**		**Full Model**	
	**Pure**	**Combined**	**Pure**	**Combined**	**Pure**	**Combined**
	ΔAUC (*p* value)		ΔAUC (p-value)		ΔAUC (*p* value)	
5-miR-panel	0.156 (0.0003)	0.0144 (0.410)	0.0534 (0.248)	0.0243 (0.259)	0.0220 (0.634)	0.0478 (0.0571)
Combined	0.170 (<0.0001)		0.0777 (0.008)		0.0698 (0.007)	

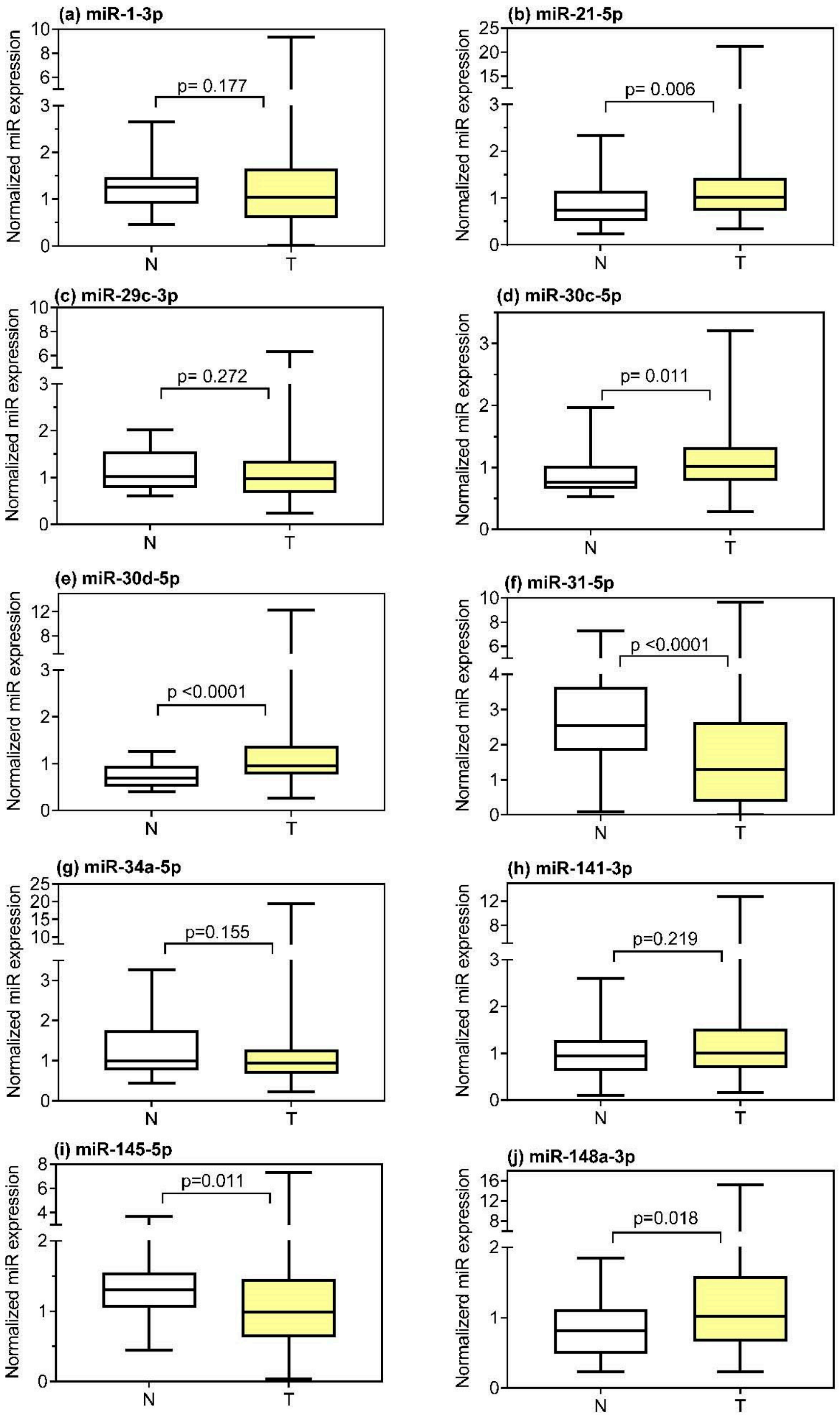
(a) miR-1-3p
Normalized miR expression
p= 0.177
N
T
(b) miR-21-5p
Normalized miR expression
p= 0.006
N
T
(c) miR-29c-3p
Normalized miR expression
p= 0.272
N
T
(d) miR-30c-5p
Normalized miR expression
p= 0.011
N
T
(e) miR-30d-5p
Normalizerd miR expression
p <0.0001
N
T
(f) miR-31-5p
Normalized miR expression
p <0.0001
N
T
(g) miR-34a-5p
Normalized miR expression
p=0.155
N
T
(h) miR-141-3p
Normalized miR expression
p=0.219
N
T
(i) miR-145-5p
Normalized miR expression
p=0.011
N
T
(j) miR-148a-3p
Normalizerd miR expression
p=0.018
N
T

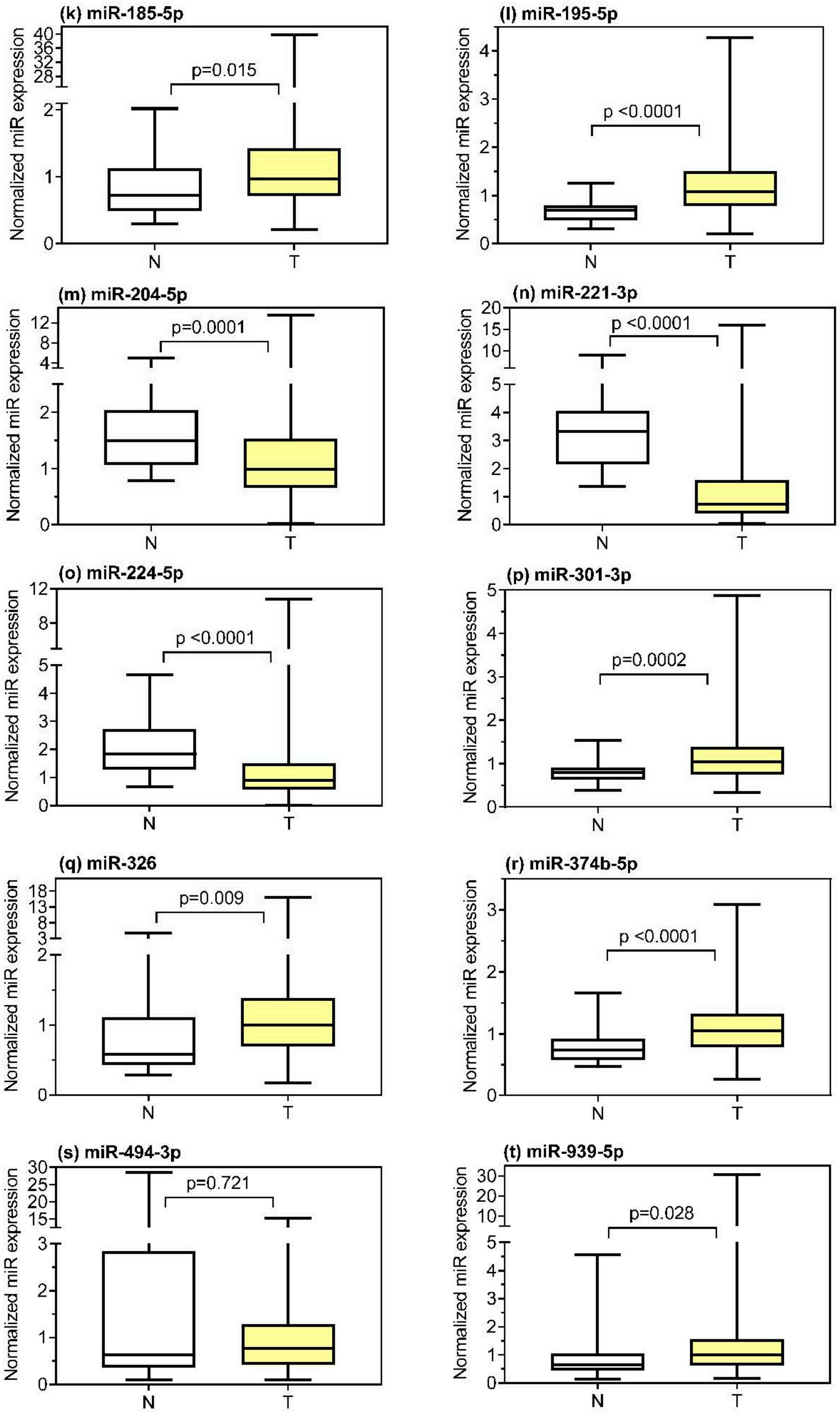

Figure S1. Expression of the 20 microRNAs analyzed in adjacent normal tissue samples (N; n=29) and all PCa tissue samples from patients with and without BCR (T; n=206). Normalized miRNA expression data are given as box- and whisker plots. Boxes represent the lower and upper quartiles with medians, whiskers illustrate the ranges of the miRNAs. Mann-Whitney *U*-test was performed. Abbreviations: Pca, prostate cancer; BCR, biochemical recurrence.

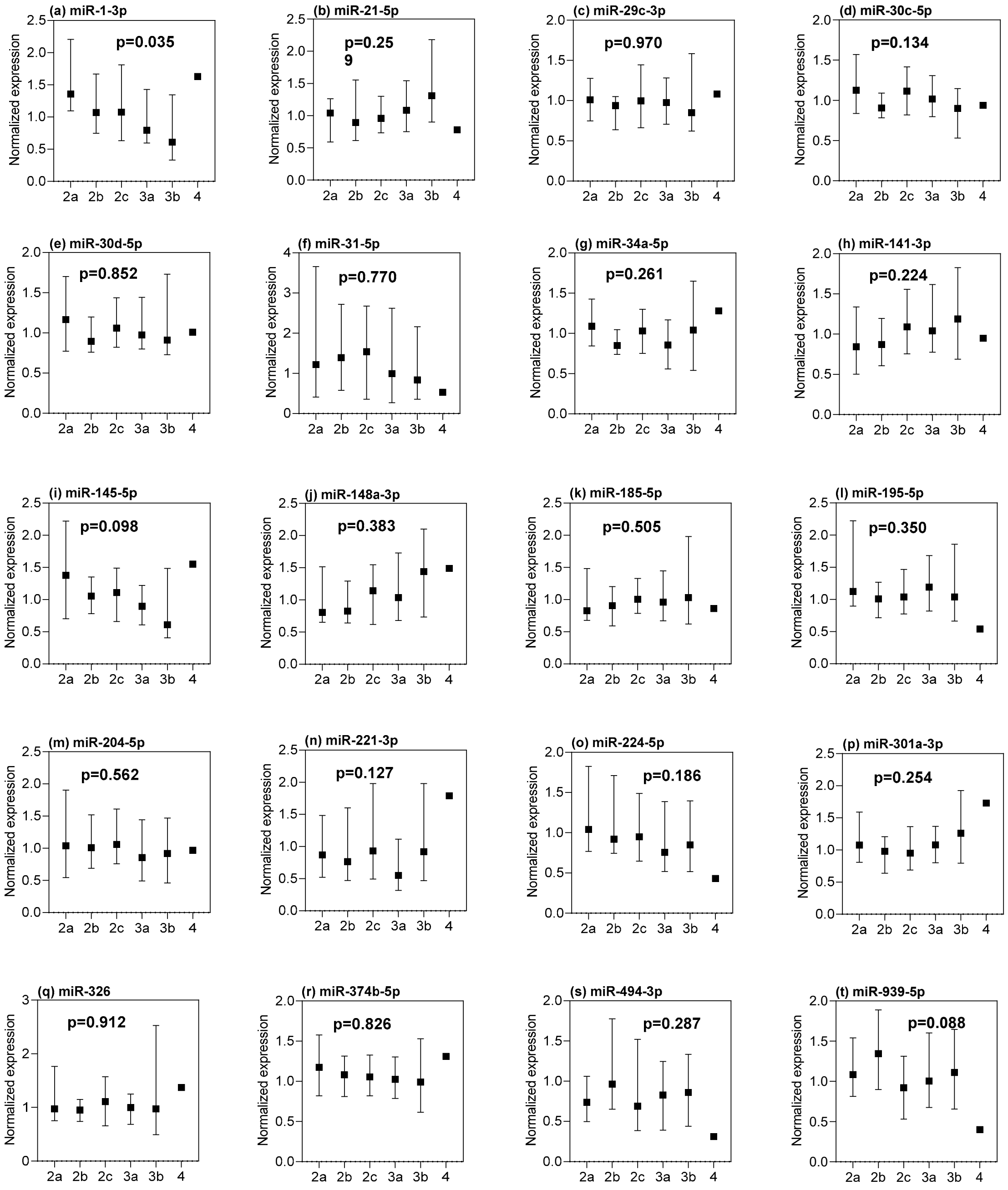

Figure S2. Expression of the 20 microRNAs analyzed in PCa tissue samples categorized according to the pT stages. Expression data are given as medians with upper and lower quartiles. Significances are calculated by the Kruskal-Wallis test. Abbreviation: PCa, prostate cancer.

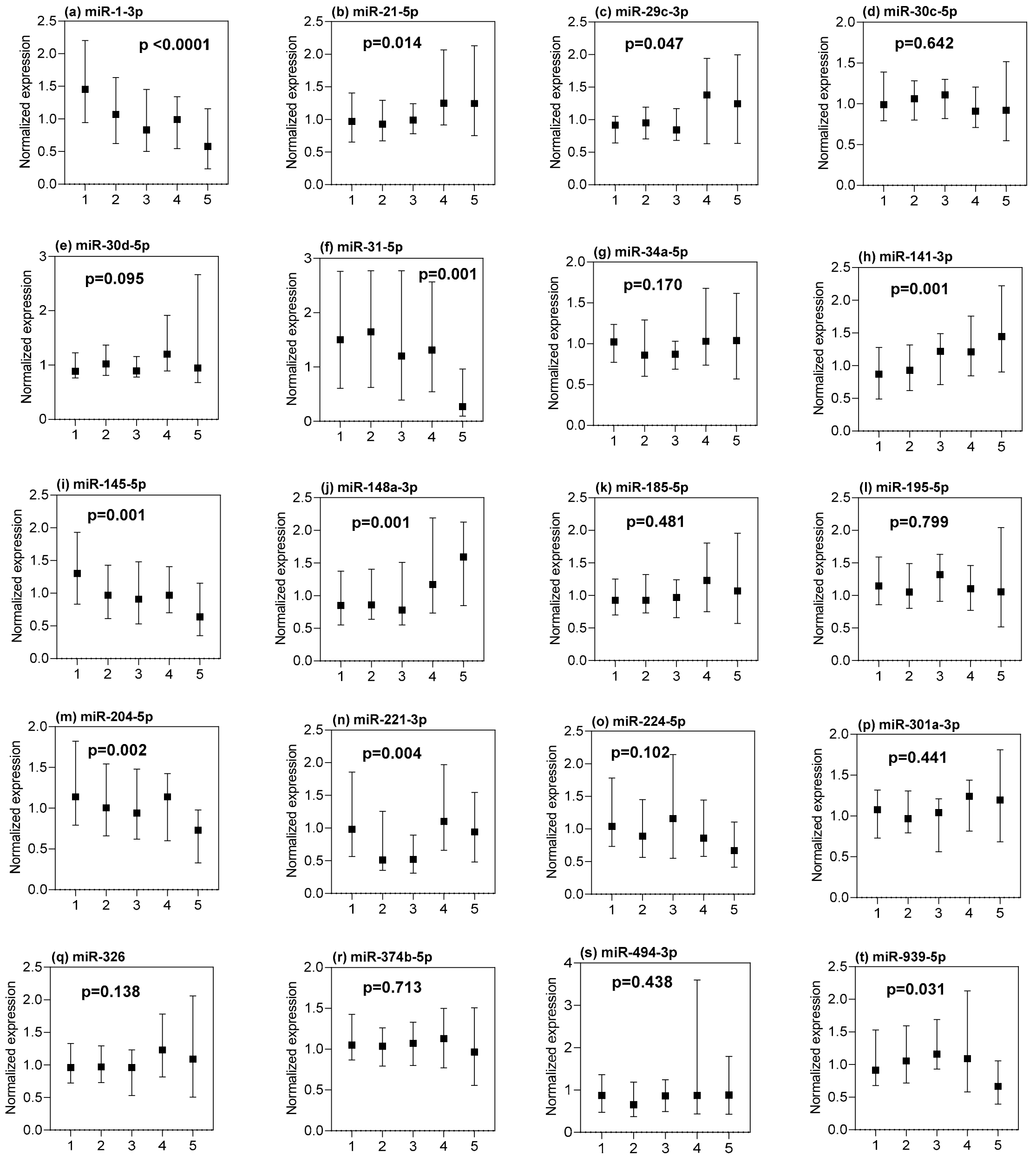

Figure S3. Expression of the 20 microRNAs analyzed in PCa tissue samples categorized according to the ISUP grades. Expression data are given as medians with upper and lower quartiles. Significances are calculated by the Kruskal-Wallis test. Abbreviation: PCa, prostate cancer.

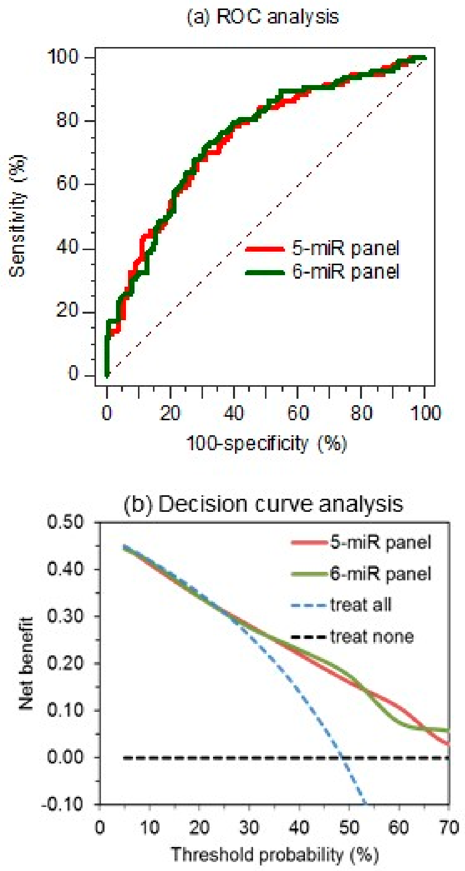

Figure S4. ROC curve and decision curve analysis of the 5-miR-panel and the 6-miR-panel. Abbreviation: ROC, receiver operating characteristics.

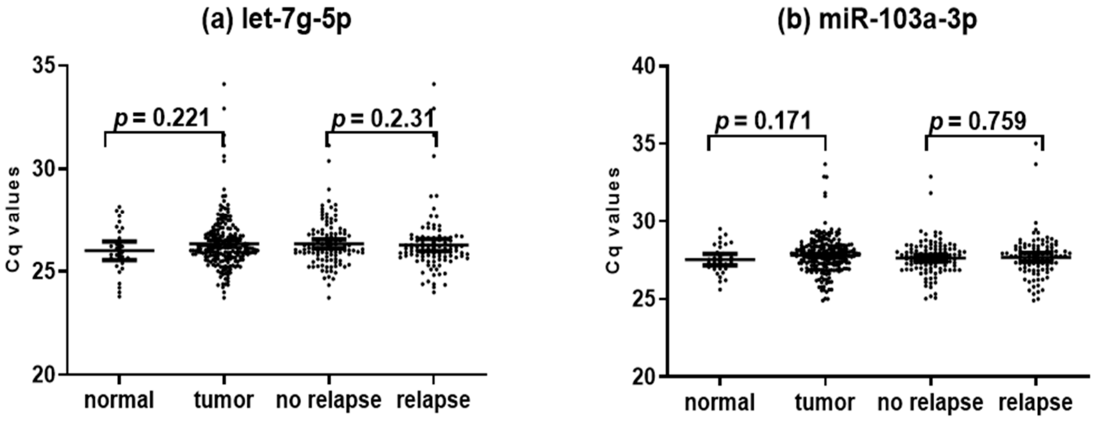

Figure S5. Stability of let-7g-5p and miR-103a-3p as suitability criterion for their use as normalizers.
